# Memory Decline and Behavioral Inflexibility in Aged Mice Are Correlated With Dysregulation of Protein Synthesis Capacity

**DOI:** 10.3389/fnagi.2019.00246

**Published:** 2019-09-04

**Authors:** Wenzhong Yang, Xueyan Zhou, Tao Ma

**Affiliations:** ^1^Alzheimer’s Disease Core Center, Department of Internal Medicine-Gerontology and Geriatric Medicine, Wake Forest University School of Medicine, Winston-Salem, NC, United States; ^2^Department of Physiology and Pharmacology, Wake Forest University School of Medicine, Winston-Salem, NC, United States; ^3^Department of Neurobiology and Anatomy, Wake Forest University School of Medicine, Winston-Salem, NC, United States

**Keywords:** aging, memory, synaptic plasticity, LTP, protein synthesis, eEF2, mTOR, Alzheimer’s disease

## Abstract

Understanding the molecular mechanisms underlying age-associated cognitive impairments will not only contribute to our general knowledge about “aging” biology, but also provide insights for more effective strategies to prevent and improve the quality of life for both normal aging and pathological aging such as Alzheimer’s disease (AD). Here we first assessed and compared the performance of cognition and synaptic plasticity in young (3–5-month old) and aged c57BL/6J mice (19–21 months old). Findings from behavioral tests demonstrated that old mice, compared to young mice, displayed impairments in spatial learning/memory, working memory, and behavioral flexibility. Further, synaptic electrophysiology experiments on hippocampal slices revealed that the early form of long-term potentiation (LTP, a synaptic model for memory formation) was inhibited in old mice. At the molecular level, biochemical assays on the hippocampus showed dysregulation of signaling pathways controlling protein synthesis capacity including: up-regulation of AKT-mTORC1-p70S6K signaling, which is associated with translation of terminal oligopyrimidine (TOP) class of mRNAs that encode translational machinery; hyper-phosphorylation of mRNA translational elongation factor 2 (eEF2) and its upstream regulator AMP-activated protein kinase (AMPK), indicating repression of general protein synthesis. Moreover, young and old mice exhibited similar brain levels of translational initiation factor 2α (eIF2α) phosphorylation, which is known to be increased in AD and linked to the disease pathophysiology. Thus, our data provide evidence at the molecular level to highlight the similarity and difference between normal and pathological aging, which may contribute to future studies on diagnostic/prognostic biomarkers for aging-related dementia syndromes.

## Introduction

Certain aspects of cognitive abilities, such as learning and memory, decline with normal aging (Burke and Barnes, 2006; Klencklen et al., 2012). With average life expectancy increasing dramatically over the last few decades and the number of the aging population is expected to triple by 2050, the prevalence of age-associated cognitive impairments grows rapidly, presenting serious public health challenges worldwide (Plassman et al., 2007; Plassman et al., 2011). Elucidation of the mechanisms underlying age-associated cognitive impairments will not only contribute to our general understanding of “aging” biology, but also provide insights for more effective strategies to prevent and improve the quality of life for both normal aging and pathological aging (e.g., Alzheimer’s disease) population (Gallagher and Rapp, 1997; Penner et al., 2010; Alzheimer’s Association, 2016).

The molecular mechanisms driving the aging-related memory decline remain unknown. Episodic/spatial and working/executive memories (associated with hippocampus and prefrontal cortex, respectively) are among those cognitive processes that are most vulnerable to advanced aging (Burke and Barnes, 2006; Foster et al., 2012; Morrison and Baxter, 2012). Moreover, multiple lines of evidence suggest that subtle morphological and/or biochemical neuronal changes, instead of profound loss of neurons, are responsible for aging-related impairments of cognition and synaptic plasticity, which is often measured in vertebrates as long-term potentiation (LTP); a synaptic model for memory (Bliss and Collingridge, 1993; Morrison and Baxter, 2012). A substantial body of evidence demonstrates that *de novo* protein synthesis (mRNA translation) is indispensable in maintaining long-lasting forms of memory and synaptic plasticity (Klann and Dever, 2004; Alberini, 2008). Of interest is that activities of translational factors involved into various stages of protein synthesis and synthesis of translational machinery *per se* (i.e., translational capacity) are known to be regulated in synaptic plasticity and memory formation by various signaling pathways. For instance, the mammalian target of rapamycin complex 1 (mTORC1) controls cap-dependent translation initiation via its downstream target eukaryotic initiation 4E binding protein 1 (4EBP1), and synthesis of translational apparatus (e.g., ribosomal proteins) encoded by terminal oligopyrimidine (TOP) class of mRNAs (Meyuhas, 2000; Tsokas et al., 2007; Hoeffer and Klann, 2010). Another molecular mechanism of mRNA translation regulation involves phosphorylation (by one of the four kinases including PERK, PKR, GCN2, and HRI) on the α subunit of initiation factor 2 (eIF2α), leading to inhibition of translation initiation and thus general protein synthesis (Wek et al., 2006; Trinh and Klann, 2013). In addition to initiation, protein synthesis is regulated at the elongation phase through multiple elongation factors, including elongation factor 2 (eEF2). Phosphorylation of eEF2 by its kinase eEF2K results in disruption of peptide growth and general protein synthesis. Potential upstream regulators of eEF2K-eEF2 includes mTORC1, and AMP-activated protein kinase (AMPK), a central molecular sensor to maintain cellular energy homeostasis (Hardie et al., 2012; Taha et al., 2013, Kenney et al., 2014).

In the current study, we assessed and compared the performance of spatial/working memory, behavioral flexibility, and hippocampal synaptic plasticity in young (3–5-month old) and aged c57BL/6J mice (19–21 months old). Further, we explored detailed brain molecular signaling mechanisms that might be associated with aging-related behavioral and electrophysiological phenotypes.

## Materials and methods

### Mice

Breeders for C57BL/6J mice were purchased from the Jackson Laboratory (Bar Harbor, ME, United States). All mice were housed in the barrier Mouse Facility at Wake Forest School of Medicine Animal Facility. Mice were kept in compliance with the National Institute of Health (NIH) guide for Care and Use of Laboratory Animals. The facility kept a 12 h light/dark cycle with regular feeding, cage cleaning, and 24 h access to water. Male and female mice (7 male and 5 female in young, 6 male and 5 female in old group), aged 3–5 or 19–21 months, were used for these experiments.

### Mouse Behavioral Testing

All behavioral tests were performed in the morning, with at least 1 h of habituation in the behavioral room prior to experimentation on each day.

#### Morris Water Maze (MWM)

Morris water maze (MWM) test was performed as described (Ma et al., 2013). The training paradigm for the hidden platform version of the MWM consisted of 4 trials (60 s maximum; interval 15 min) each day for 5 consecutive days. The probe trial was carried out 2 h after the completion of training on day 5. The visible platform task consisted of four trials each day for two consecutive days with the escape platform marked by a visible cue, and moves randomly between four locations. The trajectories were recorded with a video tracking system (Ethovision XT).

#### Novel Object Recognition (NOR)

The NOR test was performed as described with minor modification (Hoeffer et al., 2008). The test was based on the natural tendency of mice to explore a novel object rather than a familiar object. The amount of time spent exploring the novel object was normalized by the total time spent exploring both objects to yield a preference index to calculate percent object preference.

#### Reversal Y Water Maze

Reversal Y water maze test was conducted as described previously described (Hoeffer et al., 2008). At the day of training, the animal was trained to pick up one side of the maze, where a platform was hidden. The memory test phase began after a delay of 24 h, which included five trials. For mice chose the right arm, the escape platform was switched to the opposite arm, and the mice were trained to learn the new location of the platform.

### Hippocampal Slice Preparation and Electrophysiology

Acute 400 μm transverse hippocampal slices were prepared using a Leica VT1200S vibratome as described previously (Ma et al., 2014). Slices were maintained before experimentation at room temperature for at least 2 h in artificial cerebrospinal fluid (ACSF) containing (in mM) 118 NaCl, 3.5 KCl, 2.5 CaCl_2_, 1.3 MgSO_4_, 1.25 NaH_2_PO_4_, 5.0 NaHCO_3_, and 15 glucose, bubbled with 95% O_2_/5% CO_2_. For electrophysiology, slices were placed in a submersion chamber. Monophasic, constant-current stimuli (100 μs) were delivered with a bipolar silver electrode placed in the stratum radiatum of area CA3. Field excitatory postsynaptic potentials (fEPSPs) were recorded using a glass microelectrode from the stratum radiatum of area CA1. Before LTP, the input-output relationship was determined and the stimulation intensity for baseline at which the fEPSP was around 0.5–1 mV was chosen. Late LTP was induced using high-frequency stimulation (HFS) consisting of two 1-sec 100 Hz trains separated by 60 sec, each delivered at 70–80% of the intensity that evoked spiked fEPSPs. Early LTP was induced using one-train HFS (100 Hz) delivered at 25–30% of the intensity that evoked spiked fEPSPs.

### Western Blotting

Mouse hippocampus was harvested and flash-frozen on dry ice and sonicated as previously described (Ma et al., 2013). Samples containing equal amounts of protein lysate were loaded on 4–12% Tris-glycine SDS-PAGE gels for standard gel electrophoresis. Membranes were probed overnight at 4°C using primary antibodies for the following antibodies: p-Kv4.2 (1:300; Santa Cruz); Kv4.2 (1:500; Alomone Labs); GluA1 (1:1000; Cell Signaling); p-mTOR (Ser2448) (1:1000; Cell Signaling); mTOR (1:1000; Cell Signaling); p-p70S6K (Thr389) (1:1000; Cell Signaling); p70S6K (1:1000; Cell Signaling); p-4EBP1 (Thr37/46) (1:1000; Cell Signaling); 4EBP1 (1:1000; Cell Signaling); p-AKT (Ser473) (1:1000; Cell Signaling); AKT (1:1000; Cell Signaling); p-GSK3β (Ser9) (1:1000; Cell Signaling); GSK3β (1:1000; Cell Signaling); p-eIF2α (Ser51) (1:1000; Cell Signaling); eIF2α (1:1000; Cell Signaling); p-eEF2 (Thr56) (1:1000; Cell Signaling); eEF2 (1:1000; Cell Signaling); p-AMPKα (Thr172) (1:1000; Cell Signaling); AMPKα (1:1000; Cell Signaling); AMPKα1 (1:1000; Cell Signaling); AMPKα2 (1:1000; Cell Signaling); p-ERK (1:1000; Cell Signaling), ERK (1:1000; Cell Signaling), β-actin (1:5000; Sigma-Aldrich). Protein bands were visualized using chemiluminescence (Clarity^TM^ ECL; Biorad) and the Biorad ChemiDoc^TM^ MP imaging system. Densitometric analysis was performed using ImageJ. Data were normalized to β-actin (for total proteins analysis) or relevant total proteins (for phospho proteins analysis) unless otherwise specified.

### Data Analysis

Data were presented as mean ± SEM. Summary data were presented as group means with standard error bars. For comparison between two groups, a two-tailed independent Student’s *t*-test was used. Error probabilities of *p* < 0.05 were considered statistically significant.

## Results

### Impairments of Spatial Learning and Memory in Aged Mice

**FIGURE 1:**
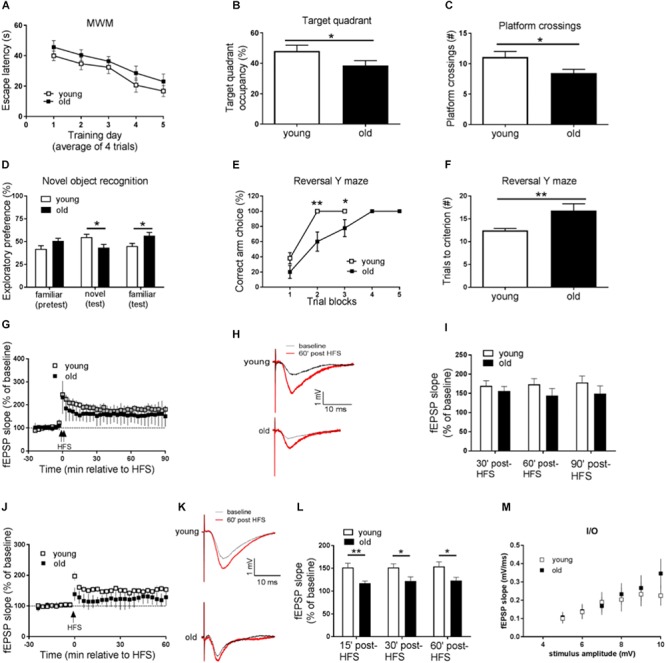
Aged mice display memory impairments, behavioral inflexibility, and synaptic plasticity failure. **(A–C)** Compared to young mice, performance of old mice on spatial learning and memory task assessed by Morris water maze (MWM) was impaired as indicated by longer day-to-day escape latency **(A)**, lower percentage of time spent in the target quadrant **(B)**, and fewer “platform” crossing frequency **(C)** during a 60 s probe trial. **(D)** Working memory performance assessed by Novel object recognition (NOR) was impaired in old mice. **(E,F)** Behavioral flexibility assessed by reversal Y water maze was impaired in old mice compared to young mice, as indicated by more training trials to locate the reversed platform. **(G)** Hippocampal L-LTP induced by 2 × HFS was similar in old and young mice. **(H)** Representative fEPSPs before and 60 min after 2 × HFS for LTP experiments shown in **G**. **(I)** Cumulative data showing mean fEPSP slope 30, 60, and 90 min after 2 × HFS based on LTP experiments shown in **G**. **(J)** Hippocampal E-LTP induced by 1 × HFS was inhibited in old mice compared to young mice. **(K)** Representative fEPSPs before and 60 min after 1 × HFS for LTP experiments shown in **J**. **(L)** Cumulative data showing mean fEPSP slope 15, 30, and 60 min after 1 × HFS based on LTP experiments shown in **J**. **(M)** Input-output relationship showed no difference between young and old mice. *n* = 12 for young mice and *n* = 11 for old mice. ^∗^*p* < 0.05, ^∗∗^*p* < 0.01.

We first tested both groups of mice on the hidden platform MWM, a spatial learning and memory task dependent on the hippocampus. Compared with the young mice, aged (old) mice displayed impaired learning and memory, as demonstrated by longer day-to-day escape latency during the acquisition phase (Figure 1A), and less target quadrant occupancy, as well as fewer “platform” crossing frequency during the probe trial (Figures 1B,C). To assess whether the impaired performance of old mice on hidden platform MWM could be attributed to memory-independent effects such as vision and swimming ability, we further tested the mice on the visible platform task, and did not observe differences between the young and old groups of mice (Supplementary Figure S1A). In addition, both young and old mice exhibited similar travel distance and average swimming velocity during the probe trial (Supplementary Figure S1B). Taken together, spatial learning and memory is impaired in aged mice.

### Working Memory Assessed by Novel Object Recognition Is Impaired in Aged Mice

Next we investigated age-related working memory alterations by performing a novel object recognition (NOR) test on the mice. As shown in Figure 1D, young mice preferred novel objects over familiar objects, as indicated by more exploration time of novel objects. On the contrary, aged mice showed enhanced preference for the familiar object as indicated by more time of explorations with the familiar object.

### Behavioral Flexibility Assessed by Reversal Y Water Maze Is Impaired in Aged Mice

To further evaluate potential cognitive alterations in aged mice, we tested the mice in Y-water maze task. Briefly, mice were trained to find an escape platform in one arm of a Y maze filled with water. Memory test demonstrated that young and old mice performed equally well in regular Y water maze task (Supplementary Figures S1C,D). The escape platform was then switched to the opposite arm, and the behavioral flexibility of the mice to learn the new location of the platform was measured (Trinh et al., 2012). Notably, compared to young mice, old mice needed significantly more trials to learn the platform switch correctly (nine out of 10 correct choices as the criteria) (Figures 1E,F), indicating impairments of their brain behavioral flexibility.

### Early LTP but Not Late LTP Is Suppressed in Aged Mice

We further examined potential aging-associated effects on hippocampal LTP, a major form of synaptic plasticity and established cellular model for learning and memory (Bliss and Collingridge, 1993). By applying weak (one train) HFS or strong (two train) HFS (see methods) protocol on acute living hippocampal slices, we induced either early LTP (E-LTP) or late LTP (L-LTP), respectively. Usually L-LTP is considered to be dependent on gene expression (translation and transcription), while E-LTP requires post-translational modification involves kinases and phosphatases (Blitzer et al., 2005). To our surprise, L-LTP was unaffected in old mice, compared to young mice (Figures 1G–I). Instead, E-LTP was significantly repressed in old mice (Figures 1J–L). These findings suggest that hippocampal short-term synaptic plasticity is impaired in aged mice. Additionally, we assessed input-output relationship by plotting stimulation intensity and fEPSP slope. No difference was observed between young and old groups (Figure 1M).

### Up-Regulation of the Brain mTORC1 Signaling in Aged Mice

**FIGURE 2:**
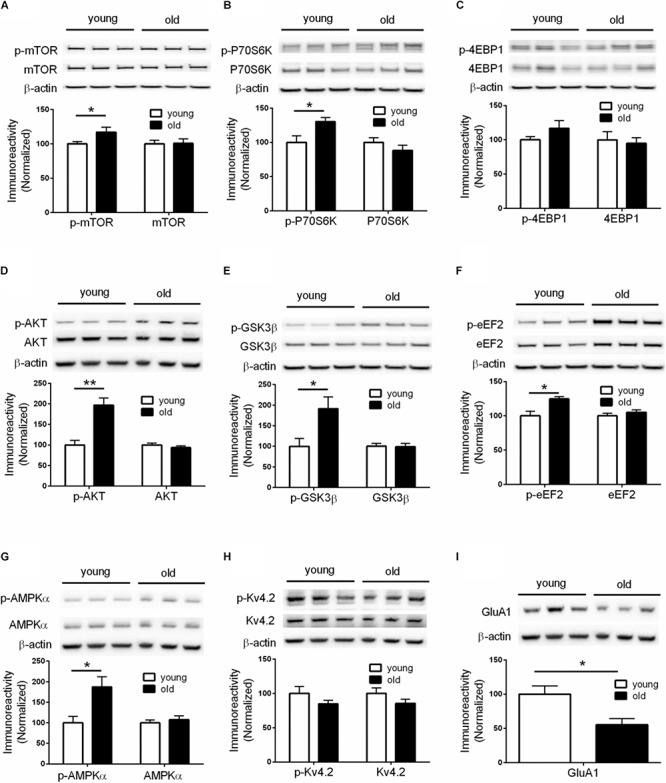
Dysregulations of protein synthesis capacity in aged mice. Western blot performed on hippocampal tissues demonstrated that compared to young mice, old mice exhibited: **(A)** Increased levels of mTOR phosphorylation (Ser2448); **(B)** Increased levels of p70S6K phosphorylation (Thr389); **(C)** Unaltered levels of 4EBP1 phosphorylation; **(D)** Increased levels of AKT phosphorylation (Ser473); **(E)** Increased levels of GSK3β (Ser9); **(F)** Increased levels of eEF2 phosphorylation (Thr56); **(G)** Increased levels of AMPKα phosphorylation (Thr172); **(H)** Unaltered levels of Kv4.2 phosphorylation; **(I)** Decreased levels of GluA1 expression. Except for GluA1, no change on levels of total proteins was observed between young and old mice. *n* = 7 for young mice and *n* = 6 for old mice (representative bands from three mice per group). ^∗^*p* < 0.05, ^∗∗^*p* < 0.01.

Given the critical role of *de novo* protein synthesis in memory formation and synaptic plasticity, we went on to investigate whether major molecular signaling pathways controlling protein synthesis are affected with aging by performing Western blotting experiments on hippocampal tissue. The mTORC1 signaling controls cap-dependent mRNA translation initiation (mediated by 4EBP1) and synthesis of translational machinery (mediated by p70S6K), and its role in memory and neural plasticity has been intensively studied (Hoeffer and Klann, 2010). Compared to young mice, brain mTORC1 signaling in old mice was up-regulated, as indicated by increased levels of phosphorylation on mTOR (Ser2448) and its downstream substrate p70-S6K (Thr389) (Figures 2A,B). Of interest, brain activity of 4EBP1 (assessed by phosphorylation at the mTOR-dependent site) was not altered in aged mice (Figure 2C). In agreement, activity of AKT, a positive upstream regulator of mTORC1 signaling (Ma et al., 2011), was increased as indicated by increased levels of phosphorylation (Figure 2D). Moreover, brain activity of GSK3β, which may exert tonic inhibition on mTORC1 signaling (Ma et al., 2011), was suppressed in aged mice, as indicated by elevated phosphorylation at Ser9 site (Figure 2E). We also examined whether there exists aging-related effects on eIF2α phosphorylation, another key translation initiation mechanism important for long-lasting forms of synaptic plasticity and memory (Trinh and Klann, 2013). Western blotting showed that levels of phospho-eIF2α (Ser51) of aged mice were indistinguishable from those of young mice (Supplementary Figure S2A).

### Elevated eEF2 Phosphorylation in Aged Mice

In addition to initiation, mRNA translation is also heavily regulated at the elongation phase through several elongation factors including eEF2. Phosphorylation of eEF2 on Thr56 prevents it from binding to the ribosome, leading to disruption of peptide growth and protein synthesis (Kenney et al., 2014). Further, a role of eEF2 phosphorylation in learning and memory has been demonstrated by many studies (Taha et al., 2013). We found that, compared to young mice, brain levels of eEF2 phosphorylation (Thr56 site) were markedly increased (Figure 2F), which is associated with a repressed capacity for general protein synthesis (Taha et al., 2013). A key upstream regulator of eEF2 phosphorylation is the central energy sensor AMPK (Hardie, 2014). We examined AMPK activity by measuring levels of phosphorylation on its kinase catalytic α subunit at Thr172 site (Hardie, 2014). Consistent with the eEF2 findings, we observed increased AMPKα phosphorylation in the brains of aged mice, compared with young mice (Figure 2G). Levels of total AMPKα, including the two isoforms of AMPKα, were not altered (Supplementary Figure S2B).

### Reduction of Brain GluA1 Expression in Aged Mice

The inhibition of E-LTP in aged mice (Figures 1J–L) indicates deficits in LTP induction mechanisms. An important molecular mechanism associated with LTP induction under physiological conditions involves phosphorylation of potassium channel Kv4.2. Kv4.2 phosphorylation by ERK results in inhibition of Kv4.2, leading to increase of dendritic excitability and enhanced LTP induction (Hoffman et al., 1997; Johnston et al., 2000, Chen et al., 2006; Schrader et al., 2006). However, we did not find any age-related changes of either Kv4.2 phosphorylation or total levels of Kv4.2 (Figure 2H). Consistently, activity of brain ERK (assessed by phosphorylation) was similar in old and young mice (Supplementary Figure S2C). We further investigated levels of GluA1 protein, an essential subunit of the α-amino-3-hydroxy-5-methyl-4-isoxazolepropionic acid (AMPA) receptors. Western blotting of hippocampal tissues demonstrated levels of GluA1 in old mice were significantly lower than those in young mice (Figure 2I), which might contribute to the E-LTP inhibition findings.

## Discussion

**FIGURE 3:**
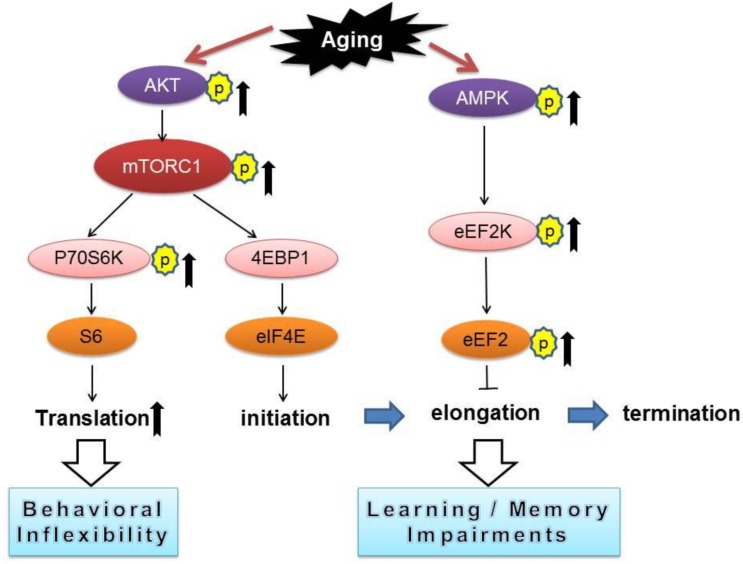
A conceptual summary of the main findings linking protein synthesis dysregulation and cognitive impairments with aging. Arrows denote activation and blunted lines indicate inhibition.

Our current study revealed that aged mice exhibited cognitive deficits (compared to young mice) assessed by a variety of behavioral tasks including: MWM (spatial learning and memory), novel object recognition (working memory), and reversal Y water maze (behavioral flexibility) (Figures 1A–F). We further observed aged-related impairments in hippocampal synaptic plasticity as indicated by impairments of E-LTP (Figures 1J–L). Importantly, biochemical assays on hippocampus from young and old mice revealed significant dysregulations of two signaling pathways controlling protein synthesis (mTORC1-p70S6K and AMPK-eEF2), indicating age-associated impairments of translational capacity (Figures 2A–G, 3).

Numerous studies have demonstrated that inhibition of general protein synthesis leads to impairments of long-lasting memory formation and failure of synaptic plasticity (Alberini, 2008; Costa-Mattioli et al., 2009). In agreement with the spatial and working memory decline in aged mice, we observed correlated hyper-phosphorylation of mRNA translation elongation factor eEF2 (Figure 2F), which causes repression of general *de novo* protein synthesis (Kenney et al., 2014). Of interest, we did not observe age-related alterations on activity of eIF2α (Supplementary Figure S2A), a key translational factor during initiation phase to control general protein synthesis (Wek et al., 2006). While it is conceivable that the initiation phase is critical for protein synthesis control, accumulating evidence points to regulation at the elongation step as an important controlling point in cellular environments such as synapses where the translational capacity is low, thus both initiation and elongation need to be boosted to accomplish the extensive requirements of new protein synthesis associated with synaptic plasticity (Tsokas et al., 2005; Sutton and Schuman, 2006).

In contrast to the findings of eEF2 hyper-phosphorylation, in aged mice we observed an up-regulation of mTORC1-p70S6K signaling (Figures 2A,B), and presumably increased protein synthesis capacity via translation of TOP mRNAs that encode components of translational machinery (Meyuhas, 2000; Tsokas et al., 2007). Of note, recent studies demonstrate that abnormal up-regulations of mTORC1 signaling, particularly on the p70S6K branch, might be the key molecular mechanism responsible for the preservative behavioral phenotype in a mouse model of autism disorder (Bhattacharya et al., 2012, 2016). Consistent with these findings, we observed impairments of behavioral flexibility in aged mice (Figures 1E,F), which might be attributed to over-activation of the mTORC1-p70S6K signaling (Figures 2A,B). Interestingly, aged mice interact more with the familiar object (compared to the interaction with novel object) in the NOR assay (Figure 1D), indicating perseverative-like, inflexible behavior (Trinh et al., 2012). Meanwhile, we did not observe dysregulations on 4EBP1 activity in old mice (Figure 2C), suggesting cap-dependent translation initiation is not affected with aging. Also worth mentioning is that in aged mice, the hippocampal E-LTP is impaired but L-LTP remains intact (Figures 1G–L). The molecular mechanisms underlying the dissociation of hippocampal L-LTP and long-term memory performance in aged mice is unclear. Data from the input-output assay indicate no difference in basal synaptic transmission between the young and old groups under our experimental conditions (Figure 1M). Of note, although it is usually considered that only “late-LTP” requires mRNA translation, protein synthesis might actually be required at a very early stage of LTP (Hernandez and Abel, 2008). Future studies are warranted to elucidate specific roles of different mRNA translational mechanisms in aging-related behavioral and synaptic plasticity phenotype.

It would be informative to compare the brain biochemical changes in normal aging and pathological aging conditions such as Alzheimer’s disease (AD). For example, brain eIF2α phosphorylation is not altered in old mice based on the current study (Supplementary Figure S2A). In contrast, hyper-phosphorylation of eIF2α has been supported by a large body of evidence to contribute to AD pathophysiology (Ma et al., 2013; Ma and Klann, 2014, Yang et al., 2016). Further, two recent studies implicate that abnormal increase of eEF2 phosphorylation (via eEF2K) is linked to AD-associated synaptic failure and neurotoxicity exerted by Aβ (Ma et al., 2014; Jan et al., 2017), and we found similar hyper-phosphorylation of eEF2 in old mice (Figure 2F). How the mTORC1 signaling is regulated in AD and its role in AD pathophysiology is subject to debate. Conflicting findings have been reported which might be due to variations such as mouse line or disease stage (An et al., 2003; Caccamo et al., 2010, Ma et al., 2010; Spilman et al., 2010, Ma and Klann, 2012), and our findings indicated age-related up-regulation of mTORC1-p70S6K signaling (Figures 2A,B). Therefore, the current study provides another piece of evidence at the molecular level to highlight the similarity (e.g., eEF2 phosphorylation) and difference (e.g., eIF2α activity) between normal and pathological aging, which may contribute to future studies on diagnostic/prognostic biomarkers for aging-related dementia syndromes.

## Data Availability

All datasets generated for this study are included in the manuscript and/or the Supplementary Files.

## Ethics Statement

Animal Subjects: The animal study was reviewed and approved by the Institutional Animal Care and Use Committee of the Wake Forest University.

## Author Contributions

WY and TM designed the experiments and wrote the manuscript. XZ and WY performed the experiments and analyzed the data.

## Conflict of Interest Statement

The authors declare that the research was conducted in the absence of any commercial or financial relationships that could be construed as a potential conflict of interest.

## Supplementary Material

FIGURE S1 **(A)** Performance of young and old mice was indistinguishable on visible platform task. **(B)** Young and old mice exhibited similar swimming distance and swimming velocity during probe trial of MWM. **(C)** Performance of young and old mice in the training phase of regular Y water maze. **(D)** Similar performance of young and old mice for the memory test of Y water maze. *n* = 12 for young mice and *n* = 11 for old mice. ^∗^*p* < 0.05.

FIGURE S2 Western blot performed on hippocampal tissues from young mice and old mice did not reveal significant changes on levels of eIF2α phosphorylation and total eIF2α **(A)**, AMPKα1 and AMPKα2 **(B)**, and ERK phosphorylation or total ERK **(C)**. *n* = 7 for young mice and *n* = 6 for old mice.
