# Supplementary figures and images for: Memory Decline and Behavioral Inflexibility in Aged Mice Are Correlated With Dysregulation of Protein Synthesis Capacity
Source: Front Aging Neurosci. 2019 Sep 4;11:246. doi: 10.3389/fnagi.2019.00246 (PMC6737270; doi:10.3389/fnagi.2019.00246)

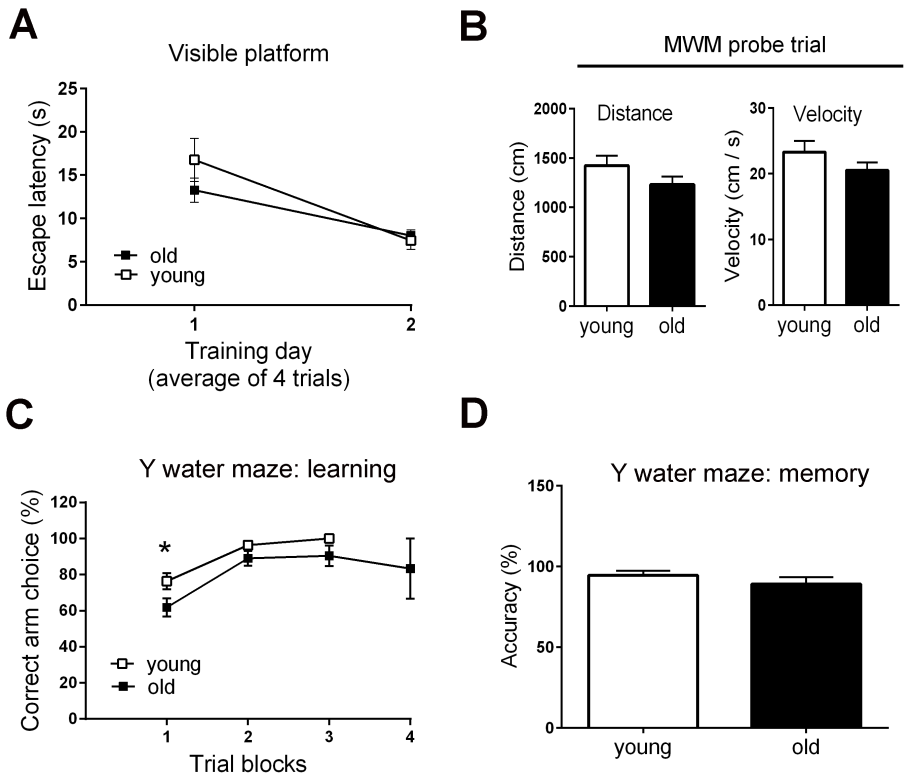

Supplement: FIGURE S1 — (A) Performance of young and old mice was indistinguishable on visible platform task. (B) Young and old mice exhibited similar swimming distance and swimming velocity during probe trial of MWM. (C) Performance of young and old mice in the training phase of regular Y water maze. (D) Similar performance of young and old mice for the memory test of Y water maze. n = 12 for young mice and n = 11 for old mice. ∗p < 0.05. [file Image_1.tif]

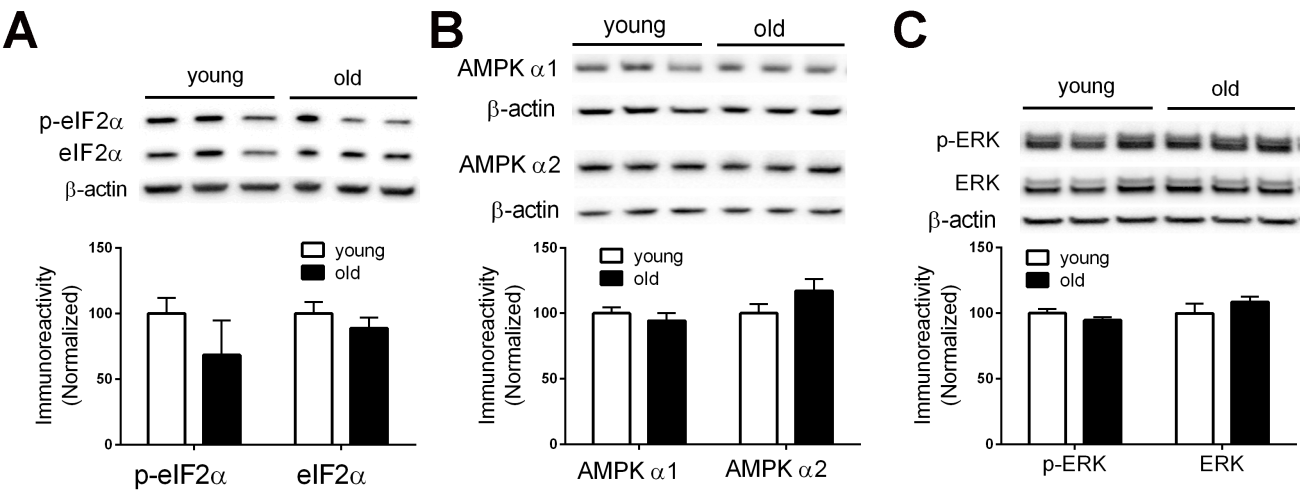

Supplement: FIGURE S2 — Western blot performed on hippocampal tissues from young mice and old mice did not reveal significant changes on levels of eIF2α phosphorylation and total eIF2α (A), AMPKα1 and AMPKα2 (B), and ERK phosphorylation or total ERK (C). n = 7 for young mice and n = 6 for old mice. [file Image_2.tif]
